# Improved physical performance of elite soccer players based on GPS results after 4 days of carbohydrate loading followed by 3 days of low carbohydrate diet

**DOI:** 10.1080/15502783.2023.2258837

**Published:** 2023-09-20

**Authors:** Abdolreza Kazemi, Ghazi Racil, Amir Hossein Ahmadi Hekmatikar, Mohadeseh Behnam Moghadam, Parisa Karami, Menno Henselmans

**Affiliations:** a Vali-E-Asr University of Rafsanjan, Dept of Sports Sciences, Faculty of Literature and Humanities, Rafsanjan, Iran; b La Manouba University, Research Unit (UR 17JS01) “Sport Performance, Health & Society” Higher Institute of Sport and Physical Education of Ksar Said, Manouba, Tunis; c Tarbiat Modares University, Department of Physical Education & Sport Sciences, Faculty of Humanities, Tehran, Iran; d Islamic Azad University, Department of Physical Education & Sport Sciences, Faculty of Humanities, Tehran, Iran; e University of Tehra, Department of Physical Education & Sport Sciences, Faculty of Humanities, Tehran, Iran; f The International Scientific Research Foundation for Fitness and Nutrition, Amsterdam, The Netherlands

**Keywords:** Exercise performance, football, nutrient periodization, carbohydrate intake, sports nutrition

## Abstract

**Background:**

Carbohydrate loading is an established sports nutrition strategy for endur- 16 ance exercise performance. We tested if carbohydrate loading could improve the performance of 17 elite soccer players under ecologically valid circumstances using Global Positioning System (GPS) data.

**Methods:**

Twenty-two adult Iran Premier league soccer players were divided into a carbohydrate-loading group (CLG) and Control group (CG). The carbohydrate loading group restricted carbohydrate intake for three days to 1.5 g/kg/d while increasing exercise intensity. From days four to seven, exercise intensity was decreased and carbohydrate intake was considerably increased up to 7.5 g/kg/d on the day of the match, during which performance was analyzed using GPS data. The control group performed the same exercise training but maintained their habitual carbohydrate intake of 5–6 g/kg/d. The data were analyzed using a univariate ANCOVA with baseline data from a pre-intervention match as the control variable.

**Results:**

The carbohydrate loading team scored significantly higher on running distance, maximum speed and the number of top and repeated sprints; the carbohydrate loading group scored significantly lower on player load, metabolic power and running imbalance compared to the control team during their match.

**Conclusions:**

Our findings suggest carbohydrate loading enabled elite soccer players to achieve greater running outputs with greater metabolic efficiency and lower fatigue compared to their habitual diets.

## 1. Introduction

Many professional soccer clubs worldwide have recognized the importance of sports nutrition and in particular nutrient periodization for athletic success [1]. Professional soccer players often classify the year into three phases with a total of around 180 training sessions: pre-season (6 weeks), in-season (39 weeks) with 30 to 60 games and off-season (7 weeks) [1]. On average, players play one match per week in-season [2,3], creating the opportunity to cycle nutrients to optimize performance on the match day, a strategy labeled carbohydrate loading [1]. Carbohydrate consumption was recognized in the early 1970s as a major energy source for soccer players [4]. Carbohydrates can be stored as glycogen in the liver (approximately 80–120 g) and muscles (approximately 350–700 g) [5]. Muscles rely on anaerobic glycolysis of their stored glycogen during high-intensity exercise, such as sprinting during a soccer match, as there is insufficient oxygen to rely purely on the aerobic system and fatty acids to provide energy sufficiently rapidly [6]. Thus, glycogen depletion from high-intensity exercise can reduce performance by lowering ATP synthesis, i.e. energy production [4,5]. High carbohydrate diets have been found to improve high-intensity exercise performance and improve muscular adaptations in athletes [7–9]. Burke et al., recommended that soccer players consume a high carbohydrate intake of 5–7 grams per kilogram of body weight per day (g/kg/d) on training days and 7–12 g/kg/d for competitions and recovery [10].

These findings have led to the development of carbohydrate loading strategies to make athletes perform better [1]. Fernandes noted that the optimal carbohydrate periodization strategy should be based on exercise intensity [11] with low carbohydrate intakes during high-intensity exercise followed by high carbohydrate intakes during low-intensity exercise to achieve glycogen supercompensation, an increase in glycogen storage levels after depletions not just back to baseline but to supra-normal levels [12]. It has been proposed that a 2–3 day decrease in carbohydrate intake with increased exercise intensity and then a decrease in exercise intensity with carbohydrate loading can result in glycogen supercompensation in athletes [13]. The findings of Bergström et al.“s 1996 study, obtained through muscle biopsy, indicated that the group which reduced their carbohydrate intake during periods of increased exercise intensity and subsequently increased their carbohydrate intake during periods of decreased exercise intensity had accumulated more muscle glycogen than the control group. Based on their results obtained from muscle biopsy, these researchers concluded that supercompensation predominantly occurs under this classic protocol [13]. ‘ Training’ low may also improve exercise adaptations. Anderson et al. reported in their study that the constant availability of carbohydrates throughout the training days reduced activity in the molecular pathways that regulate exercise adaptation [3]. Although various forms of carbohydrate periodization to improve athletes” performance have been proposed [14,15], they have not been tested in professional soccer players [1].

To further the exercise science literature and improve upon the dietary practices of soccer teams, we studied the ergogenic effects of a carbohydrate loading protocol using a GPS to track spatiotemporal data. It’s now common for elite clubs to monitor GPS metrics, such as total distance and the number of high-speed sprints [2,3,16]. Approximately 64% of the soccer teams in the European continent use GPS data [17]. Using GPS data allows coaches to design more effective training strategies and evaluate the sports performance of the players [17]. Although sports nutrition recommendations based on carbohydrate loading have been developed among athletes, recommendations applicable to soccer players are limited. According to the length of the season, football players experience different physical loads, which can be effective in creating cellular and molecular adaptations, improving performance, or reducing damage. The recommended scientific models are mostly based on muscle adaptations and post-competition recoveries, and less attention is paid to improving pre-competition performance (training sessions or days before the competition). Therefore, the practical models of carbohydrate consumption timing should be specific to the training structure (intensity, volume, and type of training). Consequently, it can be easily acknowledged that more studies are needed to fill the research gaps to advance practical recommendations for the timing of carbohydrate consumption according to the exercise structure. On the other hand, to provide simple solutions for monitoring soccer players and improving performance, using a GPS system and the timing of carbohydrate consumption can be an idea to reach better results. Therefore, in the present study, we addressed the importance of carbohydrate loading and GPS results. Despite this, it is widely acknowledged that carbohydrate loading plays a significant role in enhancing athletic performance. However, the identification of suitable strategies for carbohydrate loading in soccer players can still provide valuable insights that are in line with previous research. Therefore, this study was undertaken with the objective of investigating the importance of carbohydrate loading in improving performance. This hypothesis is linked to previous studies and contributes to further highlighting the role of carbohydrate loading in enhancing athletic performance.

## 2. Materials and methods

### 2.1. Study design

We designed a six-day carbohydrate loading intervention to examine performance-related data in soccer players during their pre-season training phase, leading up to a practice match on the seventh day. This project was registered with the code of ethics IR.RUMS.REC.1401.142 in the Rafsanjan University of Medical Sciences and with the code of Iranian Registry of Clinical Trials IRCT20221017056216N1 and ID 66,352. Before starting the training course, all subjects signed an informed consent form and participated in this study with consent and knowledge. Our inclusion criteria were: soccer players with at least five years of professional experience playing in the Premier Soccer League of Iran without any musculoskeletal injuries within the last year and who did not use any ergogenic sports supplements during the past six months.

### 2.2. Participants

To maximize ecological validity, we tracked performance during the habitual training practices of the team, including a practice match, in their training camp, so we studied 22 players (11 per team for the match).

### 2.3. Randomization

Participants were assigned an identification number and authenticated using computer-based block randomization. To ensure confidentiality, sequences were generated by one of the researchers and given to the project manager. The subjects were divided into a CLG (*N* = 11, 28.4 ± 3.0 years, 178.2 ± 5.9 cm and BMI 22.9 ± 2.3) and a CG (*N* = 11, 29.2 years ±4.19, 176 ± 4.9 cm and BMI 23.2 ± 3.2) Both groups performed the exact same training. The CG consumed their habitual diet (with 5–6 g/kg/d CHO) and the carbohydrate loading group followed a carbohydrate loading protocol. Dietary adherence was not monitored, because all players had all food provided to them in the training camp. Throughout the training protocol, all players consistently consumed the designated meals and were advised not to consume any food items that were not recommended. Also, due to the presence of the players in a training camp, they only had access to the foods that were prepared by the researchers in the camp. See Figure 2 for details.

### 2.4. Carbohydrate loading protocol

**Figure 1.**
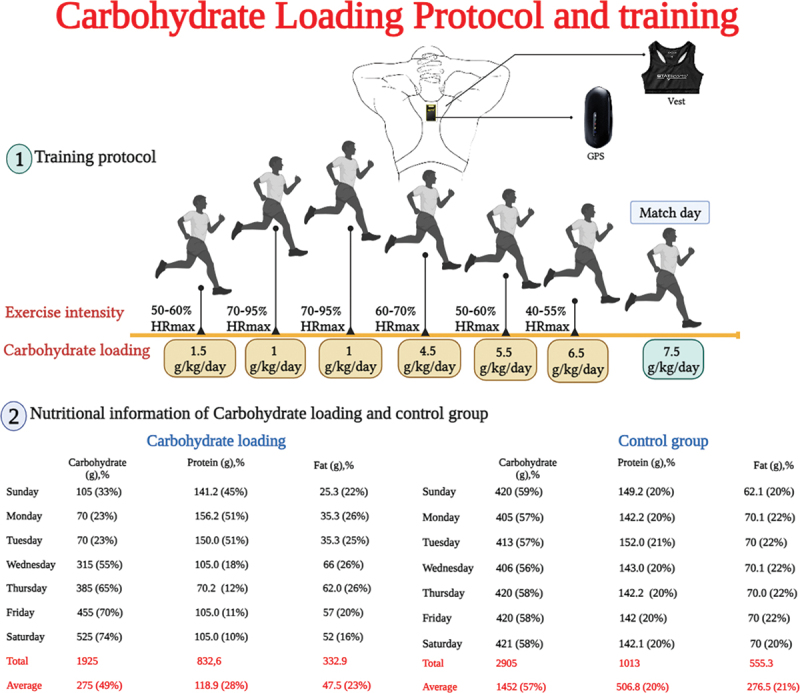
An overview of the training program and carbohydrate loading protocol.

The carbohydrate protocol consisted of three days of a low carbohydrate intake (1.5 g/kg/d on the first day followed by 1 g/kg/d on days two and three) and a higher training intensity (50–95% of maximum heart rate (HRmax)) followed by three days of progressively higher carbohydrate intake (4.5–6.5 g/kg/d) and lower training intensity (40–70% of HRmax): see Figure 1 for details. Carbohydrate intake on the match day, day seven, was 7.5 g/kg/d for the carbohydrate loading group.

**Figure 2.**
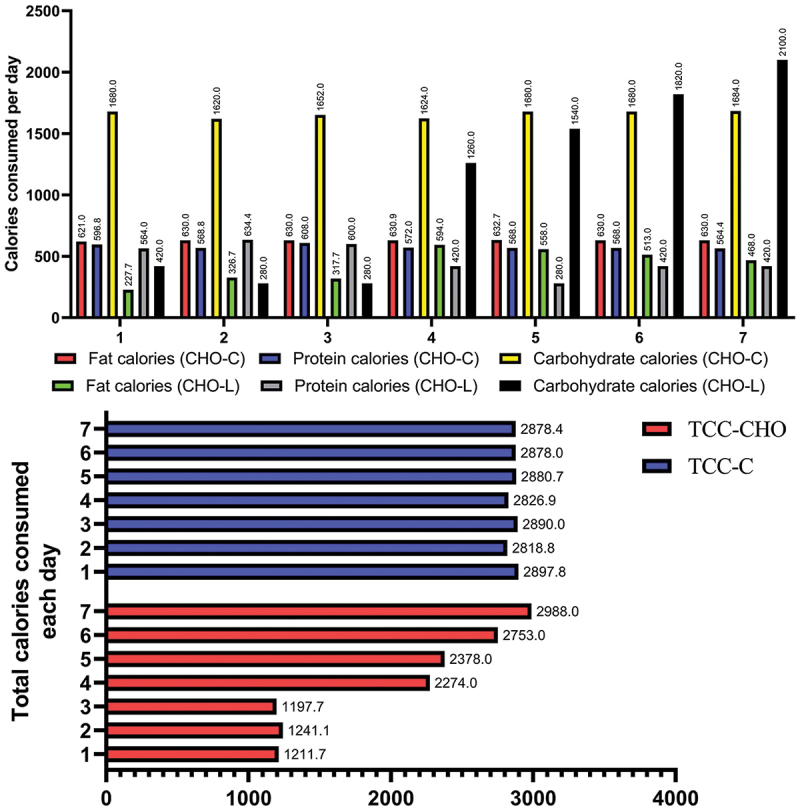
Calories consumed and total calories consumed per day. L: carbohydrate loading group. C: control group. TCC-CHO: total calories consumed in the carbohydrate loading group and TCC-C: total calories consumed in the control group.

**Table 1.** Main findings and ANCOVA *p*-values of the post-intervention between-group difference using the pre-intervention value as a control variable.

Measurements	Carbohydrate loading (pre)	Carbohydrate loading (post)	Control (pre)	Control (post)	p-value
Running distance (km)	9.96 ± 0.77	12.64 ± 1.39	10.08 ± 0.73	8.22 ± 1.13	.001*
Maximum speed (km/h)	27.02 ± 2.85	32.9 ± 4.97	28.3 ± 5.69	29.4 ± 5.77	.032*
Top sprint count (Number)	74.09 ± 11.18	121.45 ± 11.23	78.04 ± 7.58	62.06 ± 4.69	.001*
Repeated sprints (Number)	56.3 ± 11.91	81 ± 19.57	61.7 ± 5.43	60.81 ± 20.18	.001*
Player Load (kg)	301.9 ± 47.68	227.64 ± 81.56	254.5 ± 70.66	325.35 ± 128	.003*
Metabolic power (W/kg)	51.7 ± 7.61	35.8 ± 2.69	47.6 ± 5.94	41.4 ± 1.78	.001*
Running imbalance (%)	3.09 ± 0.62	0.24 ± 0.09	3.42 ± 0.79	3.52 ± 1.52	.001*

*Denotes a significant difference between the groups post-intervention.

### 2.5. Training protocol

On the first day (Sunday), the training consisted of 70 minutes of physical exercise, the intensity of which varied between 50–60% of HRmax. The training on this day included technical and tactical drills, including 4 × 4 attack and defense. The total running distance run was 2–4 km according to the GPS data. Training on Monday and Tuesday consisted of two sessions: mornings with high-intensity interval training (HIIT) and evenings with technical drills. The morning HIIT was performed at an intensity of 80–95% of HRmax: a 20 m sprint followed by a walk back to the starting position, a 40 m sprint with a walk back, etc. with 60, 80 and 100 m sprints. This five-sprint sequence was repeated five times, for a total GPS distance run of 3–5 km. The evening exercise consisted of 70 min of tactical attack and defense drills and teamwork exercises at an intensity of 70–95% of HRmax with a total GPS run distance of 7–12 km. On Wednesday, the training consisted of technical and tactical drills, such as 2 vs. 2 games, with a GPS run distance of 6–9 km at an intensity of 60–70% of HRmax. On Thursday, the training consisted of theoretical and tactical drills with a GPS run distance of 3–5 km at an intensity of 50–60% of HRmax. On Friday, the training consisted of 40 minutes of side crosses, free kicks and penalties at an intensity of 40–50% of HRmax with a GPS run distance of 2–3 km.

### 2.6. Match day measurements

The players did not have any physical activity before the match on day seven. The players had breakfast at 8:00 h and lunch at 11:00 h. The match time was set at 14:00 pm according to the Iran Premier League soccer match schedule and the match was performed according to standard league rules with two 45-minute halves and a 15-minute break, but all players wore a GPS (10 Hz) vest to enable geotracking. The results of studies have shown that GPS units (10 Hz) have the most validity and reliability for measuring the performance of athletes and football players [18–20]. It is noteworthy that all players utilized GPS devices that belonged to the brand of the smart tracking team (ST2). The players warmed up half an hour before the start of the match for 15 to 20 minutes. Due to 5 min of extra time the first half and 3 min of overtime, the total match duration was 98 min. The following measurements were recorded via the GPS for both teams:

- Average total match running distance per team. Only speeds over 3 meters per second (m/s) were considered running [21].
- The average maximum sprint speed. To evaluate the maximal sprinting speed, the maximum running speed was defined as 20 to 40 meters.
- The number of sprints with a speed over 4 m/s (top sprint count [22].)
- The number of repeated top sprints over 4 m/s within 90 s (repeated sprint count [23]).
- The Player Load (PL), defined as the amount of accumulated load a player experiences during the game based on accelerometry, as calculated below [24]. PL, also referred to as body load, was computed as a vector magnitude representing the sum of accelerations recorded in the anterior-posterior, mediolateral and vertical planes of movement, recorded by the GPS using the following equation [23]:
-
  $$\sqrt{((ax^{t=i+1}+ax^{t=i+1}){\;}^{2}}+(ay^{t=i+1}+ay^{t=i+1}){\;}^{2}+(az^{t=i+1}+az^{t=i+1}){\;}^{2})/100$$
- The Metabolic Power (MP), also called metabolic load, defined as the energy demands of acceleration and deceleration events derived from GPS, assuming a running energy expenditure of 3.6-4.0 J/kg/m [25]. The relative energy cost for accelerated running on a flat terrain can be estimated by the GPS. Subsequent multiplication with the corresponding velocity estimates the MP in W/kg. The equations provided by the GPS are as follows:
- Energy cost= (155.4ES5-30.4ES4-43.3ES3 + 46.3ES2 + 19.5ES + 36) EM
- Running imbalance: Running imbalance is the average percentage difference in load between the left and right leg when sprinting [26].

### 2.7. Statistical analysis

The data were analyzed with the software Prism 8. After confirming the normality of the data using the Shapiro-Wilk test, the univariate ANCOVA was used to analyze between-group differences in the dependent variables after the intervention. Performance during an identical test match with the same teams on their habitual diets before the intervention was used as the control variable. Comparability of the baseline data was assessed using a quantile-quantile (QQ) plot. Statistical significance was set at *p* < 0.05. The results are reported as means ± standard deviations.

## 3. Results

**Figure 3.**
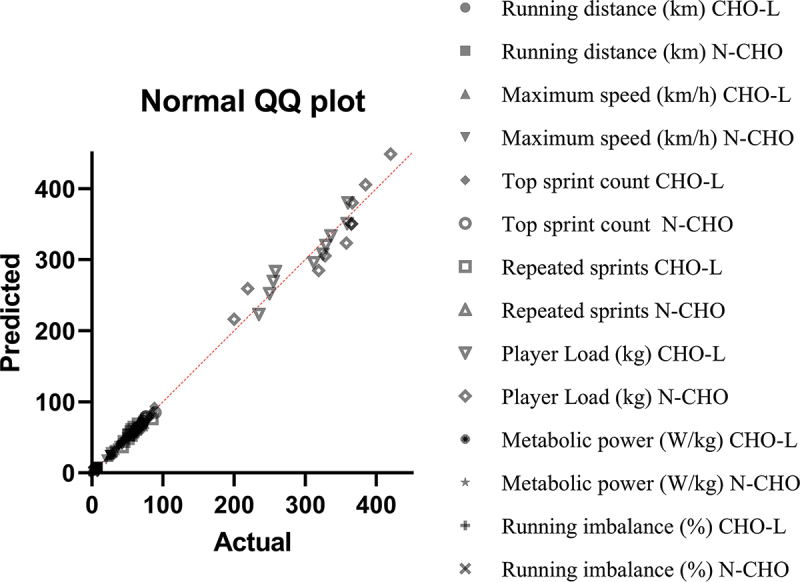
QQ plot of the performance baseline data. CHO: carbohydrate, CG: control group.

**Figure 4.**
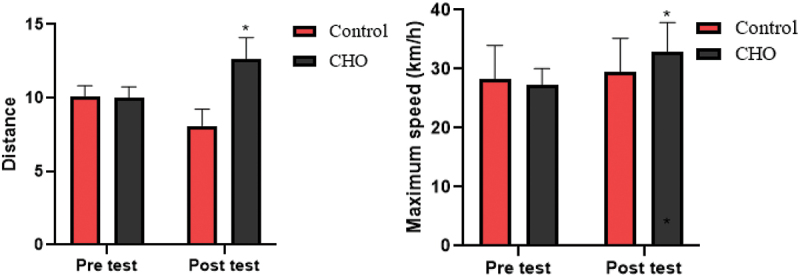
Differences in the running distance and maximum speed between groups. CHO: carbohydrate loading group.

**Figure 5.**
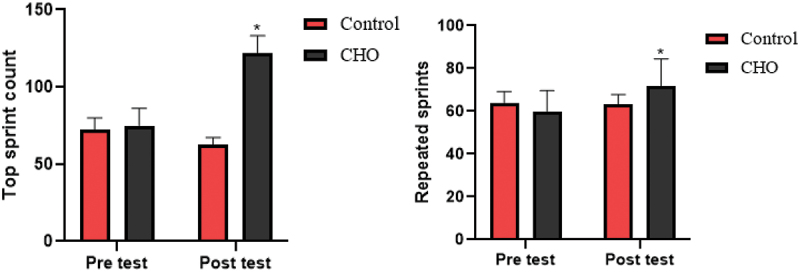
Differences in the top and repeated sprint counts between groups. CHO: carbohydrate loading group.

**Figure 6.**
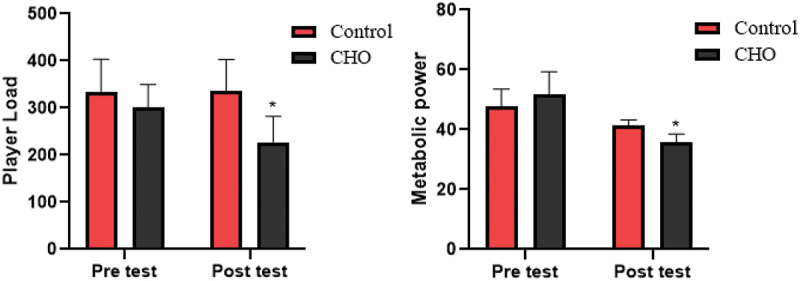
Differences in the player load and metabolic power between groups. CHO: carbohydrate loading group.

**Figure 7.**
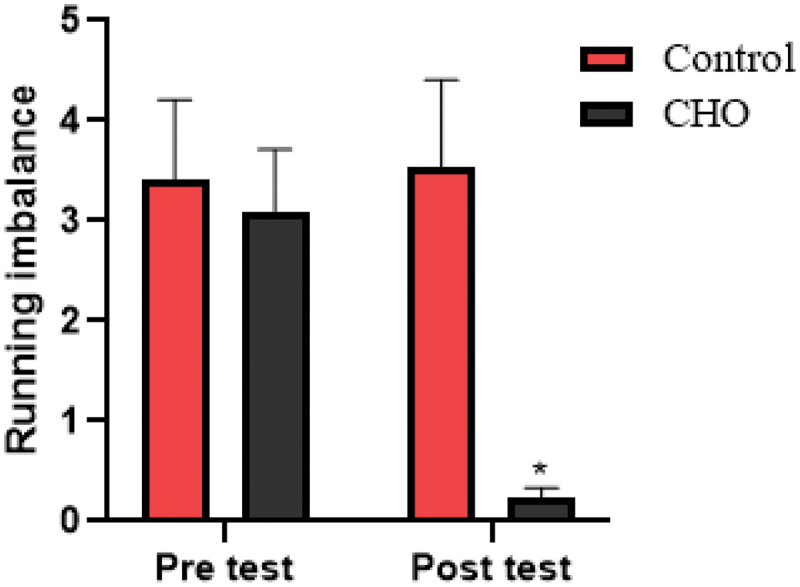
Differences in the running imbalance between groups. CHO: carbohydrate loading group.

The results of the statistical analysis aimed at assessing the significant difference between the carbohydrate loading and control groups revealed that there was a significant difference in the carbohydrate intake (*p* = .010). However, no significant difference was observed between the protein and fat consumption of the two groups (*p* = .156). Also, the results of the statistical analysis showed that there is a significant difference in total calories consumed in carbohydrates (*p* = .003), but there is no significant difference in the amounts of protein and fat between the 2 groups (*p* = .543). The baseline data were assessed as belonging to the same distribution: see the QQ plot in Figure 3. The main results are presented in Table 1 and Figures 4–7.

*Significant difference in post-intervention values.

*Significant difference in post-intervention values.

*Significant difference in post-intervention values.

*Significant difference in post-intervention values.

The results of ANOVA statistical analysis showed that there is a significant difference in running distance (F(1,17 = 25.27, *p* = .001, ES = 0.598) and maximum speed (F(1,17 = 5.48, *p* = .032, ES = 0.244) between the carbohydrate loading and control groups in the test post (see Figure 4).

The results of ANOVA statistical analysis showed that there is a significant difference in top sprint (F(1,17 = 251.267, *p* = .001, ES = 0.937) and repeated sprint counts (F(1,17 = 23.54, *p* = .001, ES = 0.581) between the carbohydrate loading group and the control group in the post test (see Figure 5).

The results of ANOVA statistical analysis showed that there is a significant difference in player load (F(1,17 = 30.04, *p* = .003, ES = 0.639) and metabolic power (F(1,17 = 26.88, *p* = .001, ES = 0.613) between the carbohydrate loading group and the control group in the post test (see Figure 6).

Finally, the ANOVA statistical analysis results showed that there is a significant difference in running imbalance between the carbohydrate loading group and the control group in the post test (F(1,17 = 128.56, *p* = .001, ES = 0.883 and Figure 7).

## 4. Discussion

We compared a carbohydrate loading protocol involving carbohydrate restriction to deplete glycogen stores followed by carbohydrate loading to achieve glycogen supercompensation to the habitual dietary practices of elite soccer players (5–6 g/kg/d carbohydrates) under ecologically valid circumstances in their training camps.

The results showed that the players in the carbohydrate-loading group consumed significantly fewer amount of calories from carbohydrates compared to the control group. However, what was surprising was that the players in the carbohydrate loading group performed better than the control group, despite both groups consuming different amounts of carbohydrates. The better performance of the carbohydrate loading group was initially highlighted by the results of running distance and maximum speed. Our reports indicate that the players in this group were able to run longer distances and reach higher maximum speeds than the control group. Our study also showed an increase in top and repeated sprint counts in the carbohydrate loading group compared to the control group, which further supports the better performance of this group. Another interesting result of our study was the reduction in player load and metabolic power in the carbohydrate-loading group. Player load and metabolic power are indicators of energy cost, and higher energy cost can lead to increased fatigue or decreased performance [27]. Our results showed that the players in the carbohydrate loading group experienced less pressure and energy cost while maintaining their performance levels. Finally, our study also showed a reduction in a running imbalance in the carbohydrate loading group, which adds validity to our results since reduced fatigue or training pressure can potentially affect unbalanced running. Overall, our findings suggest that carbohydrate loading can have positive effects on athletic performance, specifically in terms of running distance, maximum speed, and sprint counts, while also reducing energy cost and fatigue [11,28].

To our knowledge, no previous study has investigated the effect of carbohydrate loading on our study variables, so we cannot directly compare our results with previous findings. However, our findings are in line with previous research showing glycogen depletion can reduce athletic performance and high-carbohydrate diets can counteract this source of fatigue [29–32], including specifically in soccer players [33,34] utilizing carbohydrate loading [1]. For example, Bergström et al. found that glycogen storage manipulation via exercise and dieting correlated with changes in endurance exercise performance [13]. Bergström et al in their study with emphasis on supercompensation reported their results based on muscle biopsy. These researchers stated that athletes had more muscle glycogen before training after muscle biopsy. These results strongly cover the results of the present study [13]. Carbohydrates are the main fuel for high-intensity sports activities, and therefore the reduction of muscle glycogen is considered an important limiting factor for athletes [35]. Several studies have shown consumption of carbohydrates can lead to the replenishment of muscle glycogen reserves, enabling the athlete to perform high-intensity sports [35–37]. Soccer players are among the athletes who have the highest muscle glycogen depletion in type IIa and IIx fibers [36–38] and carbohydrate loading can effectively supercompensate glycogen stores [39–41]. Our findings thus support previous recommendations of high-carbohydrate diets for soccer players [42]. A soccer match may deplete glycogen stores to levels that impair contractile functioning [43], making it desirable to create as much of a glycogen buffer as possible. Carbohydrate intakes of 3–8 g/kg/d have been recommended to optimize soccer performance [1], which is in line with our finding that carbohydrate loading from 4.5 to 7.5 g/kg/d improved performance. Given that the total carbohydrate intake was higher in the carbohydrate loading group only on the day before and the day of the match, it is plausible that consuming more carbohydrates at the appropriate time, in accordance with the loading protocol (reducing training intensity and requiring more carbohydrates), and consuming the right amount of carbohydrates close to the competition may be essential for improving performance. Fernandes’ studies (2022 and 2020) have thoroughly documented the benefits of periodizing carbohydrate intake based on the intensity of training and competition [11,28]. Specifically, it is well-reported that the phasing of carbohydrate intake, based on the intensity of training and competition, should include more carbohydrates when competitions require more intensity and fewer carbohydrates when they require less intensity. This researcher reported in his results that more carbohydrate intake for competitions and less intake for training can be very effective in improving the performance of athletes. The researcher’s findings indicate that this approach can substantially improve athletes’ performance. These results align with those of the current study [11,28].

Furthermore, the present study demonstrated notable improvements in running imbalance, which exhibited a significant decrease following carbohydrate consumption. Running imbalance refers to the occurrence of slight imbalances on one side of the body resulting from factors such as fatigue, ultimately altering an individual’s gait and placing increased strain on the other side of the body [44,45]. Although the research on this specific topic is limited, the current study revealed that carbohydrate loading had the capacity to mitigate running imbalance. It is plausible that the decrease in player load and metabolic demand contributed to this favorable outcome regarding running imbalance. With caution and a degree of confidence, it can be stated that the observed reduction in running imbalance within this study can be attributed to the alleviation of fatigue and enhanced performance.

In contrast, other recent research has not found effects of acute carbohydrate intake on high-intensity running performance, running economy or critical speed [33,34,46]. In particular, Sherman et al.; found that seven days of carbohydrate loading combined with runners’ training increased glycogen reserves but did not affect their performance [30]. The discrepancy in findings could be explained by differences in statistical power, the specifics of the carbohydrate periodization protocol and the specifics of the exercise tests, along with possible adaptations to low- vs. high-carbohydrate dieting.

Our study had several notable limitations. First, we did not directly measure glycogen levels, so while previous research has shown carbohydrate loading to effectively super-compensate glycogen stores, we can not necessarily attribute the ergogenic effects of the carbohydrate loading protocol to higher glycogen stores. Future research should try to correlate the performance changes with the glycogen storage changes during carbohydrate loading. Second, the limitations of working with a real, elite soccer team in a training camp did not allow us to equate protein and energy intake between the groups, again reducing our ability to determine whether the carbohydrate manipulation per se affected performance. Third, by choosing to study performance during a match played under competitive league rules, we de facto limited our sample size to eleven per group. Given that we still found significant between-group differences, our sample size did not seem to result in prohibitively low statistical power. Fourth, since the match was played as a real match, we did not equate the player positions in both teams. Different team compositions and strategies may have confounded our results. However, by controlling for the baseline data of the same players in the same teams and using statistical significance testing, our results are highly unlikely to be solely due to chance. All study limitations arose from prioritizing external and ecological validity over internal validity. Therefore, it’s crucial that future research with a higher focus on study control and internal validity corroborates our findings.

Furthermore, this study possesses several strengths worth mentioning. First, a key strength of this study was the utilization of the GPS system as a functional tool to assess the players’ performance. This approach provided an objective measure of their performance under ecologically valid conditions. Additionally, this study addressed a gap in the existing literature concerning the significance of carbohydrate loading for elite soccer players. By introducing a novel carbohydrate loading protocol, this study showcased its potential to enhance performance in soccer players. Consequently, this new protocol became the central focal point of the article. Moreover, another strength of this study lies in the meticulous control over the players’ nutrition and sleep, ensuring standardized conditions and minimizing confounding variables.

Finally, this study showed that after reduced carbohydrate intake with increased training intensity, carbohydrate loading with doses of 4.5 to 7.5 g/per/k of body weight with reduced training intensity could be performed for four days. Therefore, in this type of strategy, we suggest carbohydrate loading with the mentioned doses as a suitable strategy to improve the performance of soccer players. Also, we tried to include carbohydrate loading in the main meal numbers throughout the day to avoid digestive issues. Considering that high carbohydrate consumption can lead to digestive problems, we did not observe any digestive issues. Finally, our study highlighted the importance of carbohydrate loading to improve the performance of elite soccer players. However, this type of carb loading was focused on soccer players, but it is thought to be effective for other sports that compete for a long time, but this needs more research to prove.

## 5. Conclusions

The current study, by highlighting the importance of super-compensation, showed that reducing the consumption of carbohydrates (to reduce muscle glycogen stores) along with increasing the intensity of training and then decreasing the intensity of training and then increasing the loading of carbohydrates can lead to an improvement in the performance of soccer players. We tried to follow the super-compensation hypothesis. In this study, the use of the GPS system as a practical tool to investigate the performance of football players played a prominent role and showed that this tool could be used for functional purposes in players. Also, this study’s consumption dose of carbohydrates showed that the highest consumption dose could be placed on the day of the game. However, the importance of this type of loading in the present study was that if a player cannot load on the day of the match, carbohydrate loading done on the previous days can be supportive. Consequently, the present study cannot definitively report the effectiveness of the loading protocol due to the inability to directly measure muscle glycogen stores. Maybe the muscle glycogen is potentially depleted and the improvement is for other reasons. However, the existing evidence indicates that carbohydrate loading is indeed effective. Furthermore, further studies are warranted to investigate various doses of carbohydrate loading for enhancing performance in soccer players, and this study builds upon previous research in this area.

## 6. Recommendations for future studies

Future research should attempt to study the effects of carbohydrate loading versus an isocaloric and isonitrogenous control group while monitoring muscle glycogen stores. This will allow researchers to determine more precisely which nutritional variables are responsible for performance and thereby optimize the dosage and timing of carbohydrate intake. It is also suggested that future studies using biopsies show the extent of muscle glycogen depletion and replenishment in a carbohydrate loading protocol. This can lead to the strengthening of the super-compensation hypothesis. In addition, our understanding of the signaling pathways is still in its infancy, so it is suggested that researchers investigate the signaling pathway of muscle hypertrophy or mitochondrial adaptations after carbohydrate loading in future studies. Also, it is suggested to investigate the importance of this type of carbohydrate loading for athletes in anaerobic disciplines such as wrestling. It is also suggested that in future studies, the relationship between running imbalance and the injuries of soccer players will be investigated through GPS.
